# Sickle Cell Anemia Treatment With Hydroxyurea in Low-Resource Settings: Challenges and Opportunities for Global North-South Collaboration

**DOI:** 10.3389/ijph.2024.1606984

**Published:** 2025-01-06

**Authors:** Teresa S. Latham

**Affiliations:** Division of Hematology, Cincinnati Children’s Hospital Medical Center Global Health Center, Cincinnati Children’s Hospital Medical Center Department of Pediatrics, University of Cincinnati College of Medicine, Cincinnati, OH, United States

**Keywords:** sickle cell anemia, capacity building, global health, hydroxyurea, pediatrics

**The IJPH series “Young Researcher Editorial” is a training project of the Swiss School of Public Health.**

Almost 8 million people suffer from sickle cell anemia (SCA), one of the most common and severe forms of inherited hemolytic anemia, and the burden of the disease is expected to substantially increase over time: by 2050, researchers estimate that 400,000 babies a year will be born with SCA, most of them in low resource settings in the Caribbean, South America, India and the Middle East and especially in sub-Saharan Africa [1]. In low-resource settings, SCA morbidity and mortality are high. Most countries with low resources have not been able to develop public health strategies to meet the growing medical burden for SCA treatment [2]. Access to comprehensive care remains limited, even though diagnosis, management, and treatment of children with SCA have drastically improved over the last decades.

Five seminal achievements offer patients with SCA new hope: 1) Prospective studies have given us a better understanding of the natural history of SCA so healthcare providers can anticipate and manage disease complications more effectively; 2) Newborns can now be screened for early diagnosis; 3) Immunizations and antibiotic prophylaxis can be administered to reduce risk of severe infection; 4) Children with SCA can undergo transcranial Doppler screening to identify those at high risk, allowing early intervention; and 5) Hydroxyurea, a disease-modifying therapy, increases fetal hemoglobin production and reduces complications. In high resource settings, children with SCA benefit from improved diagnosis, treatment, and outcomes, but in low-resource settings, progress has been inhibited by the high cost and low availability of treatments, lack of infrastructure to support regular monitoring and follow-up, and limited access to knowledge about and training in new techniques and therapies, especially hydroxyurea.

Hydroxyurea should largely replace blood transfusions in low-resource settings because it is less expensive than blood transfusion, places lower burdens on the healthcare system, does not rely on often scarce blood supplies, and poses fewer safety challenges, but it will take some time to accomplish this goal. Unlike allogeneic stem cell transplant, which is very expensive, risky, and impossible to deliver in most resource-limited setting, hydroxyurea is a once-daily oral medication that improves clinical outcomes, preserves organ function, and lowers mortality by inducing fetal hemoglobin [3]. Hydroxyurea treatment is safe and, at the maximum tolerated dose (MTD) improves clinical outcomes. But hydroxyurea is resource-intensive, since it takes 6–12 months for patients to escalate to MTD, during which time their treatment must be overseen by trained healthcare providers and their progress must be monitored with laboratory tests.

Since access to hydroxyurea in low resource settings is also limited by distribution, manufacturing, and import costs [4], and delivery is impeded by lack of training for healthcare providers who need to learn how to use hydroxyurea effectively, these challenges must also be met. In local low-resource settings, building capacity and transferring knowledge should open a path to sustainable improvement. Challenges could be addressed through collaborative efforts that build on existing capacity-building initiatives and efforts to transfer knowledge at the local level, including providing healthcare providers with comprehensive training on hydroxyurea dosing and management and improving infrastructure so it is sufficient to support regular patient monitoring and treatment oversight.

Gaps in local capacity could be affordably and sustainably filled by training and educating healthcare workers and supplementing or adding genetic counseling, newborn screening, and basic preventive care, thus strengthening the foundation upon which knowledge transfer about hydroxyurea treatment and optimal dosing must be based. All of these activities must be appropriate to the local context and be driven by a common agenda, so stakeholders and policymakers work together to leverage local strengths, build capacity through education and training, and systematically implement tools. Knowledge can also be increased in low-resource settings through prospective research, e.g., research that helps identify effective, accessible methods to administer correct hydroxyurea doses and manage patients with SCA in local settings. Qualitative researchers should help us determine the most effective methods for transferring knowledge and technology.

We can best achieve the goal of transferring knowledge and building capacity in low resource settings by leveraging North-South partnerships between groups in high-income and resource-limited countries. Successful long-term North-South partnerships must be tailored to local contexts and account for health workforce limitations in resource-limited settings. For example, a North-South partnership could resolve a difficult ethical problem by ensuring that patients with SCA who participate in a clinical trial in a low-resource setting continue to receive access to hydroxyurea after the study period ends. These partnerships would recognize that the cost of hydroxyurea is still prohibitive for most SCA patients living in resource-limited settings and could engage in structured implementation planning before the clinical trial and invest in translational research to ensure continuity of care and maintenance of clinical benefits for local patients with SCA. Partners must strategically plan technology and knowledge transfer to effectively implement change in both clinical practice and policies, and they must engage local stakeholders and implementation experts to ensure research findings are successfully transferred and translated into practice.

Prospective research on SCA treatment conducted in sub-Saharan Africa includes include the REACH and NOHARM trials, both of which documented the feasibility, safety, and benefits of hydroxyurea at MTD for African children with SCA and demonstrated the importance of global capacity-building, which facilitates knowledge and technology transfer in support of strengthening local healthcare infrastructure and informing health policy in resource-limited settings [5, 6]. In areas where access to a consistent, safe blood supply for transfusion is limited, hydroxyurea treatment lowers stroke risk and provides broader health system benefits [7, 8]. When children with SCA receive hydroxyurea treatment at MTD, their need for transfusions (including for malaria) is significantly reduced, preserving the blood supply for the general population [9]. Hydroxyurea treatment also significantly reduces transfusions when anemia is the sole indicator, likely because SCA patients treated with hydroxyurea have higher hemoglobin levels. As this knowledge is transferred, these laboratory and clinical findings should shape policy and treatment for patients, communities, and health systems burdened by low resources.

Likewise, there are existing technologies that could be transferred from high- to low-resource settings, and that could be scaled appropriately for success. One of these technologies is pharmacokinetic (PK) guided hydroxyurea dosing, which simplifies an otherwise complicated stepwise process, safely ensures doses will be optimal, and improves clinical outcomes. PK guided dosing can be based on three small-volume blood collections that are then analyzed in a laboratory; individual optimal doses can then be calculated in an online application [10]. Training for hydroxyurea PK analysis is already conducted at multiple clinical trial sites, including REACH sites, demonstrating its potential feasibility within Africa.

The benefits of hydroxyurea cannot be overstated; improving access in resource-limited settings will lead to better health outcomes for affected individuals, communities, and health systems. North-South partnerships should take steps to increase the capacity of healthcare providers and improve care delivery systems even in local contexts where healthcare infrastructure is limited cost and, where patients face accessibility barriers, and where knowledge and resource gaps exist. Collaborative efforts to provide training, facilitate access to hydroxyurea, and implement health policy to support research infrastructure will improve outcomes for children with SCA who live in sub-Saharan Africa. If we combine knowledge and technology transfer facilitated by foreign investment and North-South partnership with local engagement from policymakers, healthcare providers, and community leaders, we can open a path to sustainable hydroxyurea treatment for SCA and improve outcomes in the populations most affected by this life-threatening condition.

## Data Availability

The data analyzed in this study is subject to the following licenses/restrictions: Data supporting these results may be requested from the corresponding author of each study referenced and shared following individual study data sharing plans. Requests to access these datasets should be directed to teresa.latham@cchmc.org.
